# SARS-CoV-2 infections and MIS-C among children: A narrative review

**DOI:** 10.1097/MD.0000000000034475

**Published:** 2023-08-04

**Authors:** Ayed A. Shati, Syed Esam Mahmood, Ali Alsuheel Asseri, Ahmad A. Alhanshani, Youssef A. Alqahtani, Ausaf Ahmad

**Affiliations:** a Department of Child Health, College of Medicine, King Khalid University, Abha, Saudi Arabia; b Department of Family and Community Medicine, College of Medicine, King Khalid University, Abha, Saudi Arabia; c Department of Community Medicine, Integral University, Lucknow, India.

**Keywords:** children, coronavirus disease, multisystem inflammatory syndrome, SARS-CoV-2 infections

## Abstract

Severe acute respiratory syndrome coronavirus 2 (SARS-CoV-2) has less of an impact among the babies and teenagers, than it does on adults as a whole. Children turned out to be less symptomatic during the coronavirus disease (COVID-19) surge worldwide. Researchers discovered the ways of protection by preemptive care, like, treatment, variants, vaccination, social distancing, and cohorting among children as soon as their medical and epidemiological factors were assessed while being exposed to SARS-CoV-2 transmission. The actual pervasiveness of asymptomatic SARS-CoV-2 contagion is possibly undervalued because of less examination of the asymptomatic children. A half of young-aged people who tested SARS-CoV-2 positive don’t show any symptoms as per the study of serology. Nevertheless, there is wide circulation of information reporting a post-infectious acute illness known as multisystem inflammatory syndrome in children (MIS-C) or multisystem hyperinflammatory syndrome. Therefore, we undertook this narrative review to synthesize the evidence from existing studies to assess the relationship between SARS-CoV-2 infections and MIS-C among Children. We reviewed PubMed, Science Direct, and Google Scholar to find the pertinent scientific papers published in English that were available for such analysis. The main purpose of this article is to present, on this limited topic, a better-comprehended review covering pertinent material and data to be informed on SARS-CoV-2 infections and MIS-C among Children.

## 1. Introduction

Globally, as on 26 April 2023, there have been 764,474,387 confirmed cases of coronavirus disease (COVID-19), including 6,915,286 deaths, reported to WHO.^[1]^ As of 24 April 2023, a total of 13,343,360,939 vaccine doses have been administered. Severe acute respiratory syndrome coronavirus 2 (SARS-CoV-2) has little effect on children and teenagers, but it has affected the adults extensively causing a great number of fatalities. Despite the positive result due to lesser effect of SARS-CoV-2 on them, there still exists considerable threat of lesser manifestation infection of COVID-19 on the kids and adolescents because of lack of testing. The children or adolescents are likely to be transmitters of infection if they carry gentle signs of COVID-19. For developing, adapting, and improving control measures for COVID-19 across all ages, understanding of symptoms, infectivity, and significant SARS-CoV-2 dissemination patterns in kids and teens is required.

On the whole, comparatively the COVID-19 cases and mortality rates among the children and adolescents are substantially lesser than among the seniors. The COVID-19 cases reported to WHO based on different age groups from December 30, 2019 to September 13, 2021, kids below 5 years exhibited 5 percent of worldwide cases and 0.1 percent of worldwide mortalities.^[2]^ Of the total worldwide COVID-19 affected patients, 6.3 percent are children between the ages of 5 and 14, and of the total deaths, only 0.1 percent deaths occurred among these age groups. On the other hand, infections of younger children between the ages of 15 and 24, estimated to be 14.5 percent of the worldwide infections, and the cases of fatalities estimated to 0.4 percent of the worldwide fatalities.

The age-associated anomalies of such acuteness due to organic procedures are yet to be examined; however, the hypothetical disparities of the function and immunity of both children and adults have been addressed.^[3]^

Partially because of procedural drawbacks in the previous researches, a definitive authentication that young age is a particular risk factor for acute illness among children and adolescents is yet to be explored. Often without being hospitalized, even the children with alarming inherent health disorder, also, display the developing symptoms of SARS-CoV-2 according to numerous small-scale global researches. A protracted medical manifestation termed as (post-COVID condition or post-acute sequelae of SARS-CoV-2 infection) may be undergone by the children and adolescents with critical health condition and prolonged COVID-19 infection. Anyhow, a thorough investigation is underway to determine the frequencies and traits of these health disorders.^[4]^ The recurrence, features, and predictions of elongated indicators concerning SARS-CoV-2 conflagration cease to be certain because of restricted pursuance and lack of research with control groups. Furthermore, pediatric inflammatory multisystem syndrome temporally associated with SARS-CoV-2 (pediatric inflammatory, multisystem syndrome [PIMS-TS]) in Europe and multisystem inflammatory syndrome in children (MIS-C) in the USA are the same hyperinflammatory syndrome which is likely to make the recovery very complicated from COVID-19.^[5–8]^ In relation to the preceding derivations, for instance, cancer and immunosuppression, the intensity of illness due to new kinds of SARS-CoV-2 variants in children and adolescents is still being examined.^[9–15]^

The teenagers and their younger peers are more prone to contract SARS-CoV-2 than their elders and to transmit it unto them. This spread of infection to the juniors and vice versa depends on degree of socialization. Moreover, the initiatives carried out for restricting the microbe and organic components are concerned with the virus, for instance, the circulation of sort of version. Kids are less likely to be affected than the youngsters and teenagers; however, additional examination is required as far as the specific responsibilities of teenagers and their juniors are concerned. Generally, SARS-CoV-2 tainted juniors are much more capable of displaying gentler signs of COVID-19 health conditions. Anyway, the latest SARS-CoV-2 variants, along with the Delta, need additional examination for determining in case it does not cease to continue. Suitable preemptive initiatives, together with social isolation, hand sanitization, elbow bent or tissue coughing, indoor settings with suitable airing, and mask uses should be executed with consistency in learning centers of different age groups specifically as the youngsters below 12 years of age are not still qualified to be vaccinated to a greater extent.^[5]^ Previous reviews provide information on the association of SARS-CoV-2 infections and MIS-C among Children on a broader scale. Accordingly, the main purpose of this article is to present, on this limited topic, a better-comprehended review covering pertinent material and data to be informed on SARS-CoV-2 infections and MIS-C among Children.

## 2. Methods

This narrative review addresses the research question of what is the relationship to between SARS-CoV-2 infections and MIS-C among children. PubMed, Science Direct, and Google Scholar were the key repositories examined to find the pertinent scientific papers that were available for such analysis. The search queries, used singly and in conjunction, are “COVID-19,” “Coronavirus,” “SARS-CoV-2,” “MIS-C,” “Vaccines,” “Kids,” “Teen,” “Physical distancing,” and “Children.” Only English-language articles have been included in this narrative review (Table 1). Ethical review and approval were waived for this study due to it not directly involving human subjects (secondary research study). The study itself was a review article and did not involve human subjects and hence patient consent was waived.

**Table 1:** Methodology applied for the development of this narrative review.

Items	Specification
Date of initial search	July 10, 2020
Databases and other sources searched	PubMed, Science Direct, and Google Scholar
Search terms used	COVID-19“ OR ‘Coronavirus’ OR ‘SARS-CoV-2’ AND ‘MIS-C’ AND ‘Vaccines,’ AND ‘Physical distancing’ AND Kids,” OR “Teen,” OR “Children”
Timeframe	Studies published from June 1, 2020 to June 30, 2022
Inclusion and exclusion criteria	Observational studies and randomized control trials were considered. All literature was English language. Non-English language articles were excluded
Selection process	Search was performed after the consensus was reached between authors on relevant sources to be included

## 3. Scarce exacerbating signs in youngsters and teenagers: multisystem inflammatory syndrome in children and coronavirus disease

In April 2020, both in Great Britain and America, MIS-C is detected by doctors at Children’s hospitals. It is also termed as PIMS. A health disorder taking place after the infection of COVID-19 is known as MIS-C which affects specifically the kids of primary school background. Though the syndrome is scarce, it is very likely to be life threatening.

Expositions were published that youngsters with exceptional sickness taking after Kawasaki disorder and toxic-shock-like syndrome cropped up in the end of April 2020. As early SARS-CoV-2 symptoms were recurrently observed in these cases, the identical circumstances were also registered on both sides of the Atlantic post introductory news divulged in the UK and Italy. Thenceforth, such disease turns out to be pediatric inflammatory, multisystem syndrome momentarily connected to the infection of SARS-CoV-2 in Europe, or in America, it is called MIS-C.^[6,16,17]^

Youngsters with MIS-C tend to develop thrombocytopenia, while raised WBC count, eosinophilia, and higher platelet count are additional clues that point to the presence of Kawasaki disease. Elevated ferritin and D-dimer are more prevalent in MIS-C. Commonly older cases with MIS-C exhibits signs of psychological shock, intestinal and cardiac system, lymphopenia, and significantly rising markers levels of inflammatory. Both children above 5 (who responded with symptoms similar to those of Kawasaki disease more frequently) and younger ones emerge with coronary artery aneurysms.^[18,19]^

The average age of a second group of 186 MIS-C patients reported in a newly published study was 8.3 years, and 70% of them demonstrated PCR and serum antibody testing results for SARS-CoV-2 infection. Of the aforementioned group, 92 percent of them exhibited signs in the digestive system, 76% of them in the hematologic system, 74% of them in the mucocutaneous, 70% of them in the respiratory systems, whereas 80% of the cases needed critical care with the reports of 4 death.^[20]^ The following researches show that there are many different reported MIS-C cases and results in kids (Table 2).^[18,20–27]^

**Table 2:** A selection of pediatric studies, particularly those relating to MIS-C.

Study references	Study design	Country	Number of subjects hospitalized	COVID-19 laboratory confirmed subjects	Number of subjects with associated comorbidities	Number of subjects with MIS-C	Age (yr)	Number of subjects who died
Dufort et al^[20]^	Descriptive analyses	New York	191	95	–	95	<19 yr	2
Feldstein et al^[22]^	Retrospective surveillance	United States	186	131	–	186	<21 yr	4
Kari et al^[23]^	Retrospective analysis	Kingdom of Saudi Arabia	88	88	24	Kawasaki-like MIS-C (n = 3)	<15 yr	4
Ciftdogan et al^[24]^	Retrospective analysis	Turkey	101	62	19	101	7 yr	7
Rostami-Maskopaee et al^[25]^	Observational	Iran	225	225	73	MIS-C (n = 167) and MIS-C (n = 58) that are similar to Kawasaki	55 mo median age	10
Merckx et al^[26]^	Multicentre cohort study	Canada, San José, Costa Rica, and Iran)	232	232	50	232	5.8 yr median age	0
AlGhamdi et al^[27]^	Cross-sectional retrospective	Kingdom of Saudi Arabia	567	567	65	2	6–14 years old	4
Yousaf et al^[28]^	Surveillance investigation	USA	62	21	11	21	16 yr	0
Rhedin et al^[29]^	Cohort	Sweden	2,117,443	253	370,541	253	<19 yr	Excluded in analysis

MIS-C = multisystem inflammatory syndrome in children.

### 3.1. What is MIS-C?

Both toxic shock syndrome and a condition known as Kawasaki disease, which induce inflammation throughout the body, share characteristics with MIS-C or PIMS.

“Inflammation in the body is the first sign of the sickness after 4 weeks of SARS-CoV-2 activation. The initial warning signs and symptoms are often fever, redness, red eyes, diarrhea, and nausea. Over the following days, these symptoms could get worse. Several adolescents may become seriously unwell and require immediate medical care because the swelling can injure the cardiac organ, circulatory system, as well as other parts.”

In May 2020, CDC gave a case definition for MIS-C:

– A person with age less than 21 years, with symptoms of fever, clinically severe illness which requires hospitalization, involvement of multi-organ systems (should be more than two of the following-cardiac, respiratory, renal, gastrointestinal, hematological, neurological, or dermatological) and lab evidence of inflammation; AND
– All other diagnoses are ruled out; AND
– COVID-19 positive by a rapid antigen test, RT PCR, or serology for current or recent infection; or exposure to COVID-19 has occurred 4 weeks before the onset of symptoms.^[28]^

### 3.2. Early signs with MIS-C

If your kid has a fever and any of these symptoms, as well as an incessant temperature of more than 100 or higher enduring over 3 to 4 days, call primary care physician or pediatrician straight once:

- Unusual weakness or lightheadedness
- Having red eyes
- Redness (red patches, blotches or lumps)
- Profound or escalating stomach discomfort, vomiting, or diarrhea
- Having unusually drowsy or disoriented behavior

Despite the fact that we are still discovering more about this ailment, this is what specialists and researchers now understand:

- A somewhat rare but potentially dangerous COVID-19 complication is MIS-C. The symptoms of viruses and other illnesses could be similar. Children with symptoms should see a doctor for a screening.
- Although the condition has occasionally been observed in infants and young adults, youngsters in school-age suffer most frequently from MIS-C when they reach 8 or 9 years old.
- The onset of MIS-C symptoms typically occurs 4 weeks following a COVID-19 exposure. Antibodies to the SARS-CoV-2 are present in the majority of adolescents with MIS-C.
- Most kids who suffer from MIS-C fully recover because it is a curable illness. When used promptly, medications can reduce inflammation and prevent permanent organ damage, particularly when it involves the heart.

### 3.3. MIS-C management

If MIS-C is discovered, it can be treated. By employing drugs like intravenous fluids, steroids, and other anti-inflammatory therapy to reduce the aggravation, the hearts, renal, and other systems can be preserved from long-term impairment. There are numerous instructions used for the treatment of MIS-C, including AAP and IDSA, however there aren’t any universal ones. In order to select the best course of action, manage the symptoms, and lower the mortality rate in accordance with the management strategies outlined in (AAP) and (IDSA) standards, it is crucial to comprehend the characteristics of the disease in each patient.^[29]^

### 3.4. Children that have COVID-19 with the MIS: what connects the two?

The virus that causes COVID-19 also causes MIS-C. As a result, it can strike kids who don’t exhibit typical COVID-19 symptoms like fever, sore throat, or coughing.

According to her, the most of MIS-C sufferers may have SARS-CoV-2 antigens, which would suggest a record of body contamination. Four weeks after infectious waves with COVID-19 in that population, how many MIS-C situations there are similarly rises. Physicians and scientists are still trying to figure out why some kids get sick after contracting COVID-19 but not others. Children that have COVID-19 are susceptible to MIS-C. To avoid undesirable consequences, doctors must diagnose it with a high degree of suspicion and should begin therapy very away.^[30]^

### 3.5. MIS-C: if you have any issues, contact your physician

Physicians and scientists are now noticing that more and more youngsters do not experience serious COVID-19 indications when compared with adults. Less frequently than in cases involving adults, there have also been reports of children with the Prolonged COVID-19 issue. Prolonged COVID-19 must be distinguished from Multisystem Inflammatory Syndrome in Teenagers in this situation (MIS-C). SARS-CoV-2 infection-related symptoms that emerge or become worse over time are what define the Long COVID-19 syndrome. Researchers and medical professionals are now observing that most children typically do not have severe COVID-19 symptoms compared to adults. The long COVID-19 syndrome has also been described in pediatric patients, yet less commonly than in cases involving adults.^[31]^ Differentiating extended COVID-19 versus Multisystem Inflammatory Syndrome in Children is crucial in this situation (MIS-C). Just after intense occurrence, they continue despite necessitating a proper diagnosis.^[32]^

## 4. COVID-19 variations in young people

The delta variation has increased the number and severity of pediatric cases of COVID-19, which were previously milder in young children than in adults. Parents and other adults who are responsible for children who have the coronavirus should be aware that these kids can get sick and end up in the hospital as well as spread the virus to others.

Rarely, coronavirus-infected kids can get a serious lung infection and get COVID-19, which makes them very unwell and can even lead to death. In order to avoid infection in both adults and children, it is crucial to take precautions.

Delta forms of the coronavirus, which are more contagious, and other coronavirus variants are still spreading, especially in locations where the community COVID-19 immunization rate is low.

It is crucial to adopt tried-and-true COVID-19 measures for children who are too young to receive vaccinations (and for adults who have not had coronavirus immunizations) in order to lower the risk of contracting the virus.

Activities performed indoors are riskier than those performed outdoors, however the risk can be minimized through hand washing, masks, and better ventilation.

## 5. Infants and newborns receiving COVID-19

Rarely, mothers who have the coronavirus can infect their unborn children with it. Immediately after birth, infants might also contract an infection. Most neonates that suffer from the condition for the coronavirus have mild symptoms or none at all, and they cure, although catastrophic cases have happened. Those women who are expecting must resort to extra steps to prevent exposure to the coronavirus, such as consulting their doctor about receiving the COVID-19 vaccine.^[33]^

### 5.1. Newborns and children with COVID symptoms

In general, toddlers and babies exhibit lesser COVID-19 signs than do seniors, and perhaps some infectious youngsters may show no symptoms at all.

Children and adults who have COVID-19 symptoms include:

- Aches in the body or muscles
- Throat infection
- Change of flavor or smell
- Diarrhoea
- A headache
- Renewed exhaustion
- Nausea and vomiting
- Phlegm or a nasal congestion
- A coughing
- A temperature or shivering
- A breathing trouble or a shortness of breath

Both adults and children frequently experience fever and cough from COVID-19; adults are more likely to experience shortness of breath. Pneumonia in children can occur with or without outward signs. Additionally, they may feel diarrhea, a throat infection, or extreme weariness.

Parents should be on guard if their child is diagnosed with COVID-19 or displays symptoms because the condition can cause significant sickness in children.

## 6. Adolescent health risks from intense COVID-19

A major COVID-19 infection requiring hospitalization may be more likely in some children, according to data from the CDC study:

- Children under 2
- Due to health inequities, Black and Latino kids are more likely to have severe illnesses. A COVID-19 problem
- Young children who were born too soon
- People who have pulmonary fibrosis diseases or overweight

Trust your intuition if you suspect that your child has COVID-19, specifically if they have a congestion or even a temperature. If you are without a physician, speak to your pediatrician, healthcare professional, or emergency care facility. Pay close attention to their recommendations for confinement and screening.

## 7. Multisystem inflammatory syndrome in children

MIS-C is a health condition is given rise to those children that are exposed to the coronavirus. Doctors have discovered that after contracting the coronavirus, children may develop a disorder known as multisystem inflammatory syndrome in children, or MIS-C.

Consult your primary care physician or pediatrician right away if your child exhibits at least one of these indications and a temperature of 100.4 °F or above that persists for more than 24 hours.

- An itchy spot
- Belly (stomach) ache
- Nausea and dysentery
- Fissured, crimson lips
- Having scarlet eyeballs
- Feet and Fists with inflammation

## 8. Kids with health disorder

### 8.1. Respiratory disorder

Youngsters who are asthmatic might experience intense COVID-19 or further asthmatic illnesses, such as influenza, in comparison to children without asthma. However, keep an eye on them and, in case signs worsen consult the physicians for discussing the kids’ issues for setting up the necessary evaluation as needed. Manifestations are obvious among the majority of youngsters suffering from respiratory disorder what coronavirus has caused. Continue to replenish the prescriptions of your kids and be ready with additional precautions to keep them away from triggers for asthma attacks.

### 8.2. Diabetes

Controlling blood sugar levels is crucial. There is no reason to believe that children with well-managed diabetes will be more susceptible to COVID-19. Parents and medical professionals should be on the lookout for any manifestations in these children that may need to be evaluated because uncontrolled diabetes can impair immune function.

## 9. Children’s vaccinations

To help prevent COVID-19, everyone over the age of 5 should receive immunization against COVID-19.

- COVID-19 inoculation must be administered both for yourself and your children who are 5 years old and older, to help safeguard your entire family.
- Individuals who are not fully immunized and children under the age of five who are ineligible for the COVID-19 vaccine should continue to take precautions to stay healthy.
- Regardless of routine immunization, everyone must wear a face covering inside and outside to enhance the resistance against COVID-19, particularly its Delta version, as well as avoid the possibility of its transmission to those who are not yet afflicted.
- The only way to avoid being affected is to get vaccinated in case of having settled in densely populated area with COVID-19 infection.^[32]^

## 10. Aspects your kid’s early childhood education programme may consider

School and early childhood education (ECE) program administrators should take the following factors into account when deciding how to keep students and staff safe:

- What percentage of your local population has COVID-19 cases?
- How many people in your neighborhood have received vaccinations.
- The capacity of the community’s healthcare system to treat new patients.
- The kids’ ages in the ECE program or school.

Administrators of your school and ECE programs may alter their policies as circumstances in your community change.

### 10.1. Multiple prevention methods

The school or ECE where your child attends may employ multiple preventative strategies at once while the student is in class or participating in other activities. It’s crucial to use multiple preventative measures when some can’t be used. When people are unable to physically separate themselves from 1 another, using other preventative measures, such as face covering, and external respiration can prevent the COVID-19 dissemination.

### 10.2. Immunization against COVID-19

The best defense against developing severe COVID-19 illness is vaccination which is now at your desks for anyone aged between 5 and above, also. Discover more information on the COVID-19 vaccine for kids and teenagers.

Information on how to get immunized may be given to families by your kids’ educational institutes and their sessions of ECE. In case of any post immunization side effects, the students might be provided with flexible options for excused absences.

### 10.3. Mask utilization

Children, staff, and teachers who wear masks safeguard both themselves and those around them.

- Regardless of vaccination status, the CDC advises covering faces inside educational institutes as well as in their sessions of ECE for children 2 and above so that COVID-19 dissemination could be limited considering recent research executed on different kinds of B.1.617.2 (Delta) viruses.
- Masking of faces have been made mandatory in all kinds of transport system--even the buses carrying school children.
- Face covering is also mandatory in both transportation carrying school kids and those used for public careers. Children under the age of two should not face covering is not essential. In vehicles used for school kids, both passengers and drivers are required to wear masks, regardless of the school’s mask policy.

### 10.4. Distance and cohorting in person

There should be a physical distance rule in place at your child’s school or daycare.

While being changed, fed, and comforted by caregivers, young children must be nearby. Cohorting may therefore be used in the ECE program for your child. Children are kept together in small groups called cohorts, and each cohort stays together for the duration of the day. As a result, fewer kids and staff members interact with 1 another. Additionally, the program for your child may maximize outdoor time, space cohorts 6 feet apart, and stagger drop-off and pick-up times.

Regarding physical separation in schools, see COVID-19 Guidance for Operating Early Care and Education/Child Care Programs and Guidance for COVID-19 Prevention in K–12 Schools.

### 10.5. Testing for screening

A screening testing program might be implemented in your kid’s classroom or ECE session. Schools as well as its programs can identify COVID-19 infected individuals who may not be exhibiting symptoms by using screening tests. This makes immediate isolation easier for those who has been identified COVID-19 positive and those suspected to have come into contact with them. Find out when to put your child in quarantine.

Screening test decisions are undertaken either both in provincially and domestically. Inquire about the policies on testing, isolation, such as social distancing in the ECE program sessions and classrooms of your kids’ educational institute.

### 10.6. Air circulation

The quantity of virus-carrying airborne particles can be decreased by improving ventilation.

A school or ECE program for your child might do the following:

- By including child-safe ventilation, window opening can be made more effective.
- There must be a safe way to open multiple doors and windows for kids.
- The heating, ventilation and air-conditioning or ventilation equipment must be updated.
- It will be necessary to open or crack the windows on buses and other kinds of transportation if doing so does not put passengers at risk for injury.
- If doing so does not place passengers at danger of injury, window frames on coaches and other types of transportation must be unlocked or parted.

### 10.7. Washing hands

Instructors and administrators can utilize the ECE or school to:

- Instruct that school goers need to disinfect hands by rubbing them with sanitizers approximately for 20 seconds.
- Encourage students to frequently wash their hands and assist young children in doing the same.
- In case of difficulties in washing hands, offer disinfectant that contains approximately 60 percent ethanol. Using hand sanitizer should be done under adult supervision for young children.
- Install stations for hand washing or hand sanitizing at facility entrances.
- After handling, caring for, or feeding young children, wash your hands.
- Washing their hands both before and after handling baby bottles or changing a child’s diaper.

### 10.8. If your child is sick, keep him or her home.

Staying at home is advised if your child is ill or showing signs of COVID-19. For testing and treatment, make a call to your child’s doctor. When suffering from COVID-19, it is best to stay at home to avoid infecting others and to keep the virus out of childcare centers, schools, and other learning environments.

Understand when you should quarantine or isolate your child. As soon as the kids come back to their school being suffered from any illness, check with your child’s school or ECE program.

### 10.9. Tracking contacts

To carry out contract tracing, state and local health departments are urged to collaborate with schools and ECE programs. If the department of health approaches you regarding your child’s exposure to COVID-19, consult them to determine whether your child needs to be isolated or placed under quarantine.

### 10.10. Visiting restriction

Nonessential visitors, volunteers, and activities may be restricted by the ECE programme or your kids’ school. If a visitor is ill or exhibits COVID-19 symptoms, they should avoid entering schools.

- You might not be allowed to enter the building and would have to leave and pick up your child outside.
- There are other options for communicating with a few other mom and dad or their kids’ schoolteacher like holding outdoor or online meetings.

Access to ECE programs shouldn’t be restricted for lactating mothers.

### 10.11. Food delivery and dining

Where and when kids eat lunch may be changed by your child’s school or ECE program. For instance:

- During dining and passing through the food delivery queue, a physical distance of minimum 6 feet must be maintained because masks are removed (especially indoors).
- Changing the location of meals.
- The gym or outdoor areas are being used as extra seating areas outside the cafeteria.
- We emphasize the importance of washing hands both before and after meals for kids.
- If kids must eat indoors, they were improving the ventilation in those areas.

### 10.12. The study of physical activity and active play

Children should engage in physical play. Masks are not typically required for outdoor activities. Nevertheless, in environments with heavy COVID-19 situations, individuals now must think about donning masks when participating in crowded outdoor activities and when in close proximity to people who have not received the full course of vaccinations. Masks should be worn by both students and teachers during indoor physical education and playtime. When possible, staff members should keep a separation from the kids.

### 10.13. Extracurricular activities and sporting events

- Due to physical contact and increased breathing during some sports, participants, mentors, and instructors possibly get infected as well as disseminate the virus of COVID-19. Students and staff may also be very prone to do the same if they participate in other extracurricular activities like band, chorus, auditorium, and indoor school activities.
- Vaccinate your kid as soon as you can so they can engage in outdoor games without any dangers.
- When engaging in sports or other indoor activities, use of musk must be mandatory.
- As much as possible, games and other activities ought to be performed outside.
- When exhibiting COVID-19 symptoms, your child should get tested before engaging in these activities.
- Athletes engaged inside games and additional riskier adventures must not unmask themselves and stay away from the action as far as they can.
- If there are a lot of community cases, sporting events and exercises may be postponed or held virtually.^[33]^

## 11. How to safeguard your children against coronavirus and COVID-19

### 11.1. Get your child a COVID vaccine

There are many advantages to immunizing children against COVID-19, according to experts, including those at Johns Hopkins. Immunizations are advised for kids 5 and older, according to the CDC. Discover more information on the COVID-19 vaccine and what parents should be aware of.

### 11.2. COVID-19 prevention for young children

It is best to keep young children away from family members and other people who may be sick with the coronavirus in case they do not qualify for COVID-19 immunization. The following are the top 3 methods for preventing infections in children.

Keep a physical distance. The risk of coronavirus infection increases with the number of people and duration of contacts your children have.

- Children should keep at least 6 feet between them and people outside of their home.
- Verify that physical barriers are in place at your children’s daycare and schools (if they are accessible).
- Keep face-to-face play between kids to a minimum, and watch out for how well kids wear masks.
- Watch out that kids don’t get too close to kids or adults who are weak, like people with illnesses.

### 11.3. Putting on an appropriate mask

It is common to have the extremely contagious delta variant. Wearing a mask stops the transmission of viruses and epidemics. For this reason, even for fully immunized children in grades K–12 ought to utilize face musk as per the advices of Centers for Disease Control and Prevention and the American Academy of Pediatrics. Statistics keep proving how effective masking is at preventing the spread of infection in classrooms. Before starting back at school, Milstone advises parents to assist younger children in getting used to wearing masks so that they feel comfortable doing so.^[34]^

### 11.4. Sanitization of hands

When using the bathroom, sniffing, wheezing, or clearing noses, before consuming anything after any outdoor games, kids ought to sanitize their hands.

Milstone suggests parents to instill the habit of regular hand washing in their children, which should last at least 20 seconds. He continues the ABC song, which takes about 20 seconds to complete, may assist them maintaining a timer. In case of inaccessibility of detergent and water, Milstone believes that disinfectant with approximately 60% ethanol is the second best alternative.^[34]^

### 11.5. Hesitant youngsters

Milstone urges parents to compensate their teenagers for washing their hands by offering them a small reward, such as cards. This approach would be beneficial if your child pushes back against good hygiene or gets upset when told to do it. Don’t forget to acknowledge them for practicing good hand hygiene. By regularly washing their own hands, parents can also set a good example for their children.

### 11.6. Families and children can jointly reduce the likelihood of the coronavirus

Although COVID-19 often has a milder impact on child’s welfare than it does on grownups’, it is nonetheless essential to safeguard kids from being infected. Here’s how custodians and family members can assist.

#### 11.6.1. Take every shot you need.

Make sure that everyone in the family gets their flu shot and other vaccinations as soon as they are eligible, as well as their COVID-19 vaccinations.

#### 11.6.2. Recognize the symptoms and signs of COVID-19.

It is necessary to continue learning about COVID-19 symptoms and keeping an eye out for serious illnesses in children.

#### 11.6.3. Carefully cough and sneeze.

Milstone advises that everyone in the family use their elbows to do so rather than their hands. They should also wash their hands after doing so. He continues, “After using tissues, dispose of them.”^[33]^

#### 11.6.4. Keep your hands away from your faces.

Children ought to be reminded by their parents as little as possible to touch their faces. Toys that will keep children’s hands occupied can be helpful, according to Milstone, but guardians must sanitize those toys frequently.

#### 11.6.5. Ensure cleanliness.

Clean toys and surfaces that your child commonly touches when you are touring or while they are exposed toward a sick person. To discourage access, place cleansers goods in cabinets that are either out of your child’s reach or have childproof locks.

#### 11.6.6. How to deal with stress and anxiety.

Families can identify specific fears and establish the truth by having a discussion about the situation. Additionally, it enables households to consult a plan in case an illness or other disruption to the regular schedule occurs.^[34]^

## 12. Limitations

The information in this review as well as some significant limitations must be taken into account. Most of the data in this report came from reports that appeared early in the disease outbreak. It is still necessary for additional researchers to confirm and rely on valid and reliable methods to support many of the evident relationships mentioned in this review. To fill in knowledge gaps and find variables indicating COVID-19 problems in children, though, is crucial.

## 13. Conclusions

The assessment found a significant increase in research since the pandemic’s beginning about keeping children with COVID-19 physically apart from others, but it also identified a reliable prevention measure and looked at child protection, early care, treatment, variants, vaccination, physical separation, and cohorting. In order to improve patient outcomes, direct clinical care, and allocate scarce resources, it is crucial to identify the determinants that foretell COVID-19 issues. Further research should be addressed to analyze the occurrence of SARS-CoV-2 infections and MIS-C, its management and control among children.

## Author contributions

**Conceptualization:** Ayed A. Shati, Syed Esam Mahmood.

**Investigation:** Ali Alsuheel Asseri, Youssef A. Alqahtani.

**Methodology:** Syed Esam Mahmood, Ausaf Ahmad.

**Visualization:** Ahmad A. Alhanshani.

**Writing – original draft:** Syed Esam Mahmood, Ausaf Ahmad.

**Writing – review & editing:** Ayed A. Shati, Syed Esam Mahmood.
